# The oxidation of ABI4 by RBOHD-derived reactive oxygen species integrates redox signaling into abscisic-acid and drought-stress responses

**DOI:** 10.1016/j.abiote.2026.100037

**Published:** 2026-03-06

**Authors:** Zhenglin Ge, Tiantian Wu, Zhengyao Lin, Sisong Lai, Guojing Chen, Lihui Xiao, Jing Zhang, Kailu Zhang, Heng Zhou, Yanjie Xie

**Affiliations:** a Laboratory Center of Life Sciences, College of Life Sciences, Nanjing Agricultural University, Nanjing, 210095, China; b National Key Laboratory for the Development and Utilization of Forest Food Resources, Co-Innovation Center for Sustainable Forestry in Southern China, State Key Laboratory of Tree Genetics and Breeding, Key Laboratory of State Forestry and Grassland Administration on Subtropical Forest Biodiversity Conservation, College of Life Sciences, Nanjing Forestry University, Nanjing, 210037, China

**Keywords:** Abscisic acid (ABA), Reactive oxygen species (ROS), Sulfenylation, Stomatal closure, Drought stress

## Abstract

The phytohormone abscisic acid (ABA) induces stomatal closure and facilitates plant adaptation to drought stress. Reactive oxygen species (ROS) produced by the NADPH oxidase RESPIRATORY BURST OXIDASE HOMOLOG PROTEIN D (RBOHD) are essential components of ABA signal transduction. However, the molecular mechanisms by which ABA influences ROS production, and how ROS signaling is integrated into the ABA signaling network, are poorly understood. Here, we report that the transcription factor ABSCISIC ACID INSENSITIVE 4 (ABI4) acts upstream of RBOHD to regulate ABA-induced ROS production and stomatal closure in Arabidopsis (*Arabidopsis thaliana*). We determined that ABA-induced *RBOHD* expression and ROS production were significantly attenuated in the *abi4* mutant. In the wild type, an ABA-triggered ROS burst promoted the sulfenylation of ABI4 at Cys250, which enhanced its DNA binding and transactivation activity toward *RBOHD*, thereby regulating ABA signaling. The sulfenylation-dependent regulation of ABI4 was required for ABA-induced stomatal closure and greatly contributed to plant drought tolerance. These findings uncover a redox-dependent feedback loop in which ABI4 oxidation fine-tunes ABA responses and drought tolerance by dynamically regulating RBOHD-mediated ROS production. This post-translational regulatory mechanism for ABA signaling establishes a molecular framework explaining the crosstalk between ABA and ROS in the regulation of stomatal closure and drought resilience.

## 1 Introduction

Plants cannot escape adverse environmental conditions and must instead rely on sophisticated signaling networks to perceive and respond to environmental stress. The phytohormone abscisic acid (ABA) regulates multiple physiological processes, including seed dormancy, stomatal closure, plant growth, and responses to abiotic stresses such as drought and salinity [1]. ABA signaling is initiated by its perception by intracellular ABA receptors known as Regulatory Components of ABA Receptor/PYRABACTIN RESISTANCE1/PYR-Like proteins (RCAR/PYR1/PYL) [2]. In turn, the inhibition of PP2Cs relieves their suppression of SNF1-related protein kinase 2 (SnRK2) family members, allowing SnRK2s to be activated via phosphorylation of conserved Ser/Thr residues within their activation loops [3]. Activated SnRK2s phosphorylate downstream transcription factors, such as ABSCISIC ACID INSENSITIVE 3 (ABI3) and ABI5, as well as plasma membrane-localized NADPH oxidases (respiratory burst oxidase homologs, RBOHs), thereby promoting reactive oxygen species (ROS) production and fine-tuning ABA signal transduction [4,5].

ROS are important second messengers in guard cell signaling pathways that control stomatal movement in response to diverse stimuli [6]. In the context of ABA signaling, ROS stimulate ABA biosynthesis or inhibit ABA degradation, resulting in elevated endogenous ABA levels. In turn, ROS production is induced by ABA signaling, forming a tightly interconnected regulatory circuit [7]. Moreover, both intracellular and apoplastic ROS function at multiple steps within the ABA signaling cascade and play central roles in signal amplification during stomatal closure [8]. ROS generated by RBOHs are indispensable for ABA signal transduction [9]. Ten annotated or putative NADPH oxidase-encoding genes, *RBOHD–J*, have been identified in Arabidopsis (*Arabidopsis thaliana*). Among these, *RBOHD* is crucial for ROS generation [10]. ABA-induced ROS accumulation is highly dependent on increased *RBOHD* transcript levels and enhanced protein activity [11,12]. However, despite the central importance of RBOHD in ABA-induced ROS signaling, the mechanism by which ABA upregulates *RBOHD* expression remains poorly understood.

The signaling functions of ROS, particularly hydrogen peroxide (H_2_O_2_), largely depend on their ability to mediate oxidative post-translational modifications (OxiPTMs) of cysteine residues in their target proteins [13]. Such cysteine-based redox modifications play crucial roles in diverse physiological processes by modulating the activity, stability, and interaction capacity of key signaling proteins, including transcription factors that directly regulate gene expression [[14], [15], [16]]. The initial reaction of H_2_O_2_ with thiol (-SH) forms sulfenic acid (-SOH), which is intrinsically unstable and easily converted to other forms, such as sulfinic (-SO_2_H) and sulfonic (-SO_3_H) acids in response to excessive H_2_O_2_ exposure [17]. The initial oxidation product of the Cys residue, in its sulfenic acid (-SOH) form, is considered to be an integral part of ROS-sensing pathways, as it leads to further redox-based modifications that affect protein structure and function [18,19]. However, despite the well-known crosstalk between ABA and ROS in regulating stomatal closure and stress responses, how RBOHD-mediated ROS signals regulate ABA signaling is still unclear.

ABSCISIC ACID INSENSITIVE 4 (ABI4) was initially identified as a positive regulator of ABA signaling based on its role in ABA-responsive gene expression and developmental regulation [20]. Subsequent research has revealed its role as a multifunctional transcription factor extensively involved in crosstalk with other plant hormones, the regulation of plant developmental phases, and abiotic/biotic stress responses [14,21,22]. ABI4 regulates the expression of its target genes by binding to Coupling Element 1 (CE1)/G-box-related motifs (CACCG) or CCAC elements in their promoters [14,21]. Although *Arabidopsis* *abi4* mutants exhibit altered ABA responsiveness during vegetative growth, including defects in stomatal regulation, the underlying molecular mechanisms linking ABI4 to ABA signaling in guard cells remain unclear [14,23]. Notably, a recent study showed that ABI4 directly binds to the *RBOHD* promoter and activates its transcription [21].

Here, we aimed to carry out a comprehensive analysis integrating ABI4-mediated transcriptional regulation of *RBOHD*, subsequent ROS dynamics, and their combined impact on stomatal movement. We identified a redox-controlled ABI4 module that links RBOHD-derived ROS to ABA-induced stomatal closure and drought-stress responses. We demonstrated that ABI4 is required for ABA-induced *RBOHD* transcription and the subsequent ROS burst that governs ABA responses in *Arabidopsis*. Furthermore, ABA-triggered ROS production leads to the sulfenylation of ABI4 at Cys250, which enhances its DNA-binding affinity and transactivation activity toward RBOHD. This redox-dependent enhancement of ABI4 activity promotes further ROS accumulation, thereby reinforcing ABA-induced stomatal closure and ultimately improving plant drought tolerance. Together, our findings establish a molecular framework for the role of the crosstalk between ABA and ROS in regulating stomatal movement and drought resilience and lay the foundation for developing strategies for improving drought tolerance in crops through molecular breeding.

## 2 Results

### 2.1 ABI4 is required for ABA-induced RBOHD activation and ROS production

To gain insight into ROS dynamics in *Arabidopsis* in response to ABA, we monitored ROS contents in plants using H_2_DCF-DA staining. In wild-type Col-0 plants, ROS levels began to increase after 30 min of ABA treatment, reached a peak at approximately 60 min, and subsequently declined over time (Fig. 1A). Consistent with the previous finding that RBOHD plays a critical role in ABA-induced ROS production, ROS levels in the *rbohd* mutant did not significantly fluctuate following ABA treatment. Moreover, *RBOHD* transcript levels rapidly increased within 15 min of ABA treatment, declined sharply by 90 min, and increased again at later time points (Fig. 1B). As transcriptional activation precedes protein accumulation, the dynamic changes in *RBOHD* expression closely paralleled the observed pattern of ROS production (Fig. 1A). These results indicate that RBOHD is responsible for ABA-induced ROS production.

**Fig. 1:**
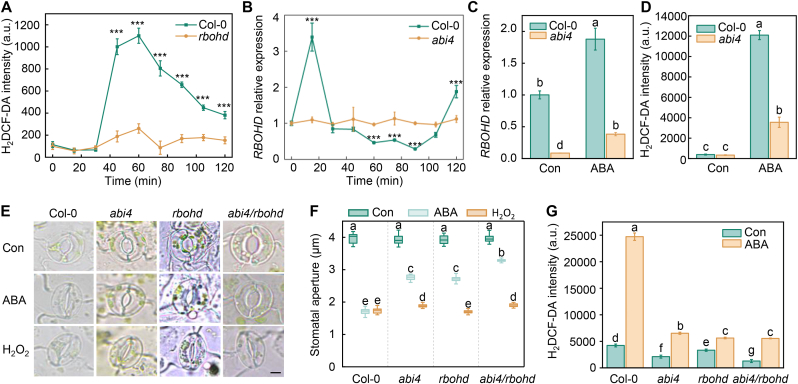
ABI4 acts upstream of RBOHD to regulate ABA-induced ROS production and stomatal closure. **A** Time-course analysis of ROS accumulation in protoplasts isolated from *Arabidopsis* Col-0 and *rbohd* mutants following treatment with 10 μM ABA. After ABA treatment for the indicated times, protoplasts were stained with 1 μM H_2_DCF-DA for 30 min, and ROS levels were quantified based on fluorescence intensity. **B** Time course results of the expression profiles of *RBOHD* in Col-0 and *abi4* protoplasts in response to ABA treatment. **C** The expression level of *RBOHD* in Col-0 and *abi4* mutants after 20 min ABA treatment. Seven-day-old Col-0 and *abi4* seedlings grown on 1/2 MS medium were treated with 10 μM ABA for 20 min, followed by RT-qPCR analysis of *RBOHD* expression. **D** ROS accumulation in leaves of Col-0 and *abi4* plants after treatment with 10 μM ABA for 30 min, as determined by H_2_DCF-DA fluorescence intensity. **E** Representative images of stomatal apertures in Col-0, *abi4*, *rbohd*, and *abi4 rbohd* mutants under control conditions or after 30 min treatment with 10 μM ABA or 10 μM H_2_O_2_. **F** Quantification of stomatal aperture in (E**)**. **G** Relative ROS levels in guard cells under control and ABA treatment conditions corresponding to (E**)**. Data represent means ± SE from at least three independent biological replicates. Asterisks denote significant differences between treatments as determined by *t*-tests (∗*P* < 0.05, ∗∗*P* < 0.01, ∗∗∗*P* < 0.001). Different letters indicate statistically significant differences (*P* < 0.05, Duncan's multiple range test).

ABI4 directly activates *RBOHD* transcription by binding to the CCAC *cis*-element in its promoter [21]. To determine whether ABI4 is involved in the ABA-mediated transcriptional regulation of *RBOHD*, we first compared *RBOHD* transcript levels in *Arabidopsis* Col-0 and *abi4* plants under ABA treatment. Under normal growth conditions, *RBOHD* transcript levels were significantly higher in Col-0 than in *abi4* (Fig. 1C). After 30 min of treatment with 10 μM ABA, *RBOHD* expression increased significantly in both Col-0 and *abi4* plants. However, the ABA-induced upregulation of *RBOHD* was significantly attenuated in *abi4*. These results indicate that ABI4 participates in the ABA-induced transcriptional regulation of *RBOHD*. We next assessed whether ABI4-dependent regulation of *RBOHD* expression is reflected at the level of ROS accumulation. ABA treatment significantly increased ROS levels in the leaves of both Col-0 and *abi4* plants. However, ROS levels in leaves under ABA treatment were significantly higher in Col-0 than in *abi4* (Fig. 1D), which is in line with the *RBOHD* expression patterns. Collectively, these results demonstrate that ABI4 is responsible for ABA-induced *RBOHD* expression and ROS production.

To further dissect the genetic relationship between ABI4 and RBOHD in regulating ABA responses in *Arabidopsis* guard cells, we generated the *abi4 rbohd* double mutant. Mutating either *ABI4* or *RBOHD* impaired ABA-induced stomatal closure (Fig. 1E and F). Importantly, this ABA-hyposensitive guard cell phenotype was more pronounced in the *abi4 rbohd* double mutant than in the single mutants. Moreover, compared to WT, H_2_O_2_ treatment fully induced stomatal closure in *rbohd*, but not in *abi4* or *abi4 rbohd* plants. These results, combined with the finding that ROS levels were similar in *rbohd* and *abi4 rbohd* plants under ABA treatment (Fig. 1G), suggest that ABI4 is responsible for ABA-induced *RBOHD* expression and ROS production and that the function of ABI4 in the ABA response is partially dependent on RBOHD.

### 2.2 RBOHD-produced ROS mediate ABI4 sulfenylation

Given that ABI4 is known to function as a redox-sensitive transcription factor [14], we examined whether ABI4 might be regulated by H_2_O_2_ through OxiPTMs. We transiently expressed a construct encoding ABI4-GFP fusion protein in *abi4* protoplasts, immunoprecipitated ABI4-GFP, and detected oxidized ABI4 using an antibody specific for cysteine sulfenic acid. Following treatment with low concentrations of H_2_O_2_ (10 and 50 μM) for 30 min, the sulfenylation level of ABI4 increased markedly. By contrast, exposure to higher H_2_O_2_ concentrations (100 and 200 μM) resulted in a pronounced decrease in ABI4 sulfenylation (Fig. 2A), indicating that ABI4 undergoes H_2_O_2_-dependent oxidative modification in a concentration-dependent manner. A similar biphasic response was observed in a time-course analysis of H_2_O_2_ treatment. ABI4 sulfenylation increased during the early phase of H_2_O_2_ exposure and subsequently declined with prolonged treatment (Fig. 2B). Notably, ABI4 remained predominantly in a monomeric form under all H_2_O_2_ treatment conditions (Fig. S1), excluding the possibility that the observed decrease in sulfenylation resulted from disulfide bond formation. Instead, the reduction in sulfenylation at higher H_2_O_2_ concentrations or longer treatment durations could likely be attributed to the overoxidation of cysteine residues, whereby sulfenic acid is further converted into sulfinic or sulfonic acid forms [17].

**Fig. 2:**
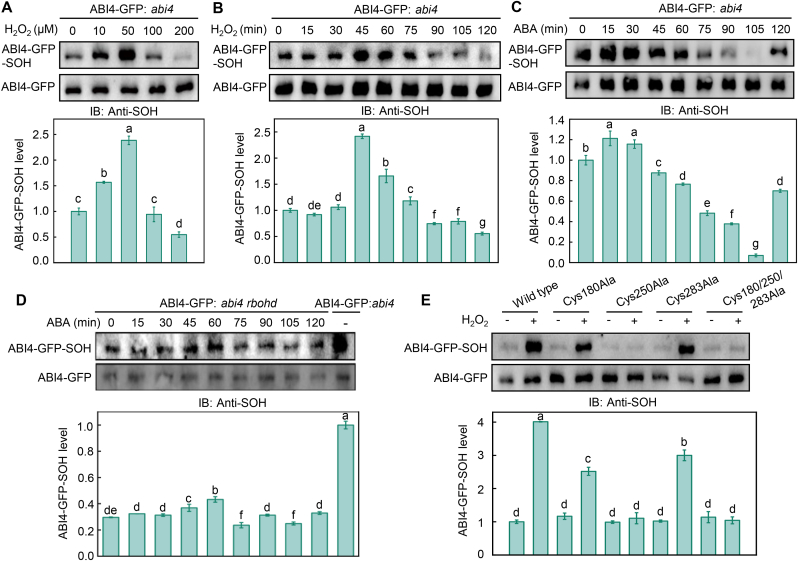
RBOHD-produced H_2_O_2_ mediates ABI4 sulfenylation. **A** Effect of H_2_O_2_ concentration on ABI4 sulfenylation. Protoplasts from *abi4* mutants transiently expressing *ABI4-GFP* were treated with the indicated concentrations of H_2_O_2_ for 60 min. ABI4-GFP was immunoprecipitated using anti-GFP magnetic beads, and sulfenylation (SOH) levels were detected using an anti-SOH antibody. **B** Time-dependent sulfenylation of ABI4 following treatment with 10 μM H_2_O_2_. **C-D** ABA-induced sulfenylation of ABI4 in protoplasts from *abi4* (C) and *abi4 rbohd* (D) mutants transiently expressing *ABI4-GFP* following treatment with 10 μM ABA for the indicated times. **E** Identification of the sulfenylated cysteine residue in ABI4. Protoplasts transiently expressing *ABI4-GFP* or cysteine-mutated variants were treated with H_2_O_2_, followed by immunoprecipitation and SOH detection as described above. Sulfenylation levels were normalized to the untreated control (set to 1.0). Data represent means ± SE from at least three independent biological replicates. Different letters indicate statistically significant differences (*P* < 0.05, Duncan's multiple range test).

To determine whether ABI4 sulfenylation occurs under physiological ABA signaling conditions, we examined ABI4 oxidation in response to ABA treatment. We transiently transfected *abi4* protoplasts with a construct encoding *ABI4-GFP*, treated the protoplasts with ABA, and detected sulfenylated ABI4. The sulfenylation level of ABI4 increased within the first 15 min of ABA exposure, followed by a gradual decline, reached a minimum at approximately 105 min, and increased again at 120 min (Fig. 2C), indicating the biological relevance of ABI4 sulfenylation in ABA signaling. Importantly, the temporal profile of ABI4 sulfenylation closely resembled the ABA-induced changes in ROS accumulation and *RBOHD* transcript abundance (Fig. 1A and B), suggesting that RBOHD-derived ROS might be responsible for ABI4 oxidation.

To investigate this hypothesis, we measured the sulfenylation level of ABI4 in *abi4 rbohd* protoplasts transiently expressing *ABI4-GFP* in response to ABA treatment (Fig. 2D). Surprisingly, the changes in sulfenylation level of ABI4 were attenuated in *abi4 rbohd* compared to *abi4* protoplasts and similar to the changes in ROS levels in *rbohd* protoplasts (Fig. 1A). These results indicate that ROS produced by RBOHD are responsible for the ABA-triggered sulfenylation of ABI4.

To identify the cysteine residue(s) responsible for ABI4 sulfenylation, we examined the sulfenylation levels of ABI4 variants carrying site-directed cysteine mutations. ABI4 contains three cysteine residues (Cys180, Cys250, and Cys283). Replacing Cys180 and Cys283 with alanine (Ala) did not affect the H_2_O_2_-induced sulfenylation of ABI4 (Fig. 2E), whereas mutating Cys250 to Ala or simultaneously mutating all three Cys abolished the H_2_O_2_-induced sulfenylation of ABI4, indicating that Cys250 is the critical residue required for H_2_O_2_-induced sulfenylation of ABI4.

### 2.3 Sulfenylation of Cys250 enhances the DNA binding ability and transactivation ability of ABI4

To explore the functional consequences of ABI4 sulfenylation, we examined whether the DNA-binding capacity of ABI4 was affected by sulfenylation by performing electrophoretic mobility shift assays (EMSAs) using a 54-bp DNA fragment containing the CCAC motif from the *RBOHD* promoter. Consistent with previous reports, recombinant ABI4 protein bound to this promoter fragment *in vitro* (Fig. 3A). Notably, ABI4 binding activity was markedly enhanced following 30 min of H_2_O_2_ treatment, whereas prolonged exposure to H_2_O_2_ (105 min) almost completely abolished its DNA-binding activity (Fig. 3B). These results, combined with the changes in sulfenylation levels of ABI4 observed under H_2_O_2_ treatment (Fig. 2B), indicate that sulfenylation enhances the DNA-binding ability of ABI4 *in vitro*, whereas overoxidation may compromise this activity.

**Fig. 3:**
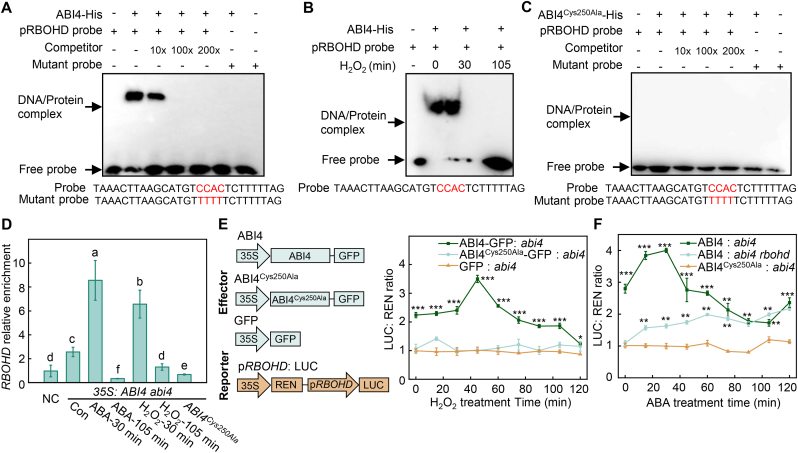
Sulfenylation of Cys250 enhances ABI4 DNA binding and transactivation activity. **A, C** Electrophoretic mobility shift assays (EMSAs) showing binding ability of ABI4 or cysteine-mutated ABI4 proteins to *RBOHD* promoter fragments containing the CCAC motif. **B** Effect of H_2_O_2_ treatment on ABI4 binding to the *RBOHD* promoter in EMSA assays. Recombinant ABI4-His proteins were treated with 10 μM H_2_O_2_ for the indicated times prior to incubation with biotin-labeled probes. **D** Chromatin immunoprecipitation followed by qPCR (ChIP-qPCR) analysis of ABI4 enrichment at the *RBOHD* promoter after 10 μM ABA or H_2_O_2_ treatment for the indicated times. A region without CCAC motif from the *RBOHD* promoter was chosen as the negative control (NC). **E** Dual-luciferase reporter assays showing the effect of H_2_O_2_ treatment on ABI4-mediated transactivation of the *RBOHD*. ABI4-GFP and ABI4^Cys250Ala^-GFP constructs were co-transfected with the *RBOHD-LUC* reporter into protoplasts of *Arabidopsisabi4* mutants. After treatment with 10 μM H_2_O_2_, dual-luciferase activity was assayed. **F** ABI4 regulates ABA-responsive *RBOHD* transcription via Cys250 sulfenylation. ABI4-GFP and ABI4^Cys250Ala^-GFP constructs were co-transfected with the *RBOHD-LUC* reporter into protoplasts of *Arabidopsisabi4* mutants. Additionally, ABI4-GFP and RBOHD-LUC were co-transfected into protoplasts of *abi4 rbohd* double mutants. After treatment with 10 μM ABA, dual-luciferase activity was assayed. Data represent means ± SE from at least three independent biological replicates. Asterisks denote significant differences between treatments as determined by *t*-tests (∗*P* < 0.05, ∗∗*P* < 0.01, ∗∗∗*P* < 0.001). Different letters indicate statistically significant differences (*P* < 0.05, Duncan's multiple range test).

To examine whether sulfenylation affects the DNA-binding ability of ABI4 *in vivo*, we conducted chromatin immunoprecipitation followed by quantitative PCR (ChIP-qPCR) using promoter fragments that were identified as ABI4 targets in a previous study [21]. ABI4 was enriched at the *RBOHD* promoter, and its binding strength closely correlated with its sulfenylation status. Specifically, 30 min of both ABA and H_2_O_2_ treatments significantly enhanced ABI4 occupancy at the *RBOHD* promoter, whereas prolonged ABA or H_2_O_2_ treatment (105 min) markedly reduced ABI4 binding (Fig. 3D). These results demonstrate that sulfenylation enhances the DNA-binding ability of ABI4. Moreover, the results of *in vitro* and *in vivo* analyses highlight the essential role of Cys250 in ABI4 function, as mutating Cys250 abolished the binding of ABI4 to the *RBOHD* promoter (Fig. 3C and D).

To investigate whether the transcriptional activity of ABI4 is regulated by its oxidation, we performed dual-luciferase assays by transiently transfecting *Arabidopsis* protoplasts with the effector construct *Pro35S:ABI4-GFP* and the reporter constructs *ProRBOHD:LUC*. The expression of *ABI4-GFP*, but not *GFP* from the *Pro35S:GFP* vector, increased LUC activity (Fig. 3E). Furthermore, LUC activity derived from the *RBOHD* promoter increased following short-term H_2_O_2_ treatment (within 45 min) and then declined during prolonged treatment of up to 120 min, which was also correlated with changes in ABI4 sulfenylation levels under H_2_O_2_ treatment (Fig. 3E). These observations indicate that the transcriptional activity of ABI4 is regulated by its sulfenylation level.

To further explore this notion, we examined the transcriptional activity of ABI4 in response to ABA treatment using the *abi4* mutant to avoid the influence of basal *ABI4* expression. As we expected, LUC activity increased in response to ABA treatment within 30 min, declined until 105 min, and increased again at 120 min, which is consistent with the changes in ABA-triggered sulfenylation of ABI4 (Fig. 3F). Moreover, the transcriptional activity of ABI4 was significantly inhibited in *abi4 rbohd* compared to *abi4*, further confirming the essential role of RBOHD-produced ROS in the sulfenylation-mediated enhancement of ABI4 transcriptional activity (Fig. 3F). However, the enhanced transcriptional activity of ABI4 triggered by both H_2_O_2_ and ABA was abolished in protoplasts transiently expressing the ABI4Cys250Ala mutant protein, which is consistent with the results of EMSA and ChIP-qPCR. These findings indicate that Cys250 plays a major role in the transcriptional function of ABI4, likely by mediating redox-dependent conformational changes that influence its DNA-binding and transactivation capacity. Collectively, these results demonstrate that sulfenylation of Cys250 is essential for the enhanced DNA-binding ability and transcriptional activity of ABI4 in ABA signaling.

### 2.4 RBOHD-produced ROS regulate ABA responses via the sulfenylation of ABI4 at Cys250

**Fig. 4:**
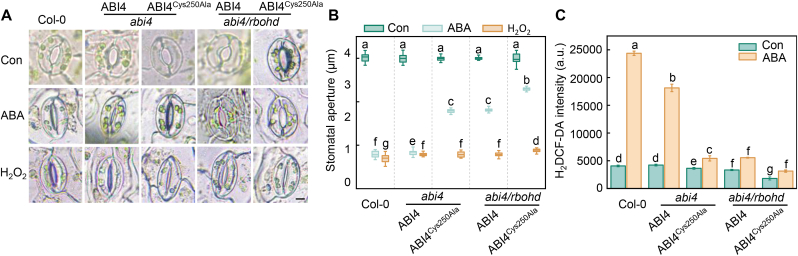
RBOHD-produced ROS controls ABA responses through sulfenylation of ABI4 at Cys250. **A** Representative images of stomatal apertures in Col-0 and transgenic lines expressing *ABI4* or *ABI4*^*Cys250Ala*^ in *abi4* or *abi4 rbohd* backgrounds under control conditions or after 10 μM ABA or H_2_O_2_ treatment for 30 min. **B** Quantification of stomatal aperture shown in (A**)**. **C** Relative ROS levels in guard cells from (A**)**. Data represent means ± SE from at least three independent biological replicates. Different letters indicate statistically significant differences (*P* < 0.05, Duncan's multiple range test).

To further investigate whether the ABA response regulated by ABI4 sulfenylation is mediated by RBOHD, we overexpressed *ABI4* and its *ABI4*^*Cys250Ala*^ variant in the *abi4* and *abi4 rbohd* backgrounds and isolated homozygous transgenic lines. The introduction of wild-type *ABI4,* but not *ABI4*^*Cys250Ala*^ into the *abi4* mutant background completely restored ABA- and H_2_O_2_-induced stomatal closure (Fig. 4A and B), suggesting that Cys250 and its sulfenylation are essential for the role of ABI4 in regulating ABA responses in terms of stomatal closure. Moreover, treatment with H_2_O_2_, but not ABA, induced stomatal closure in *35S*:*ABI4 abi4 rbohd* transgenic plants, indicating that RBOHD-associated protein sulfenylation is involved in ABA-mediated activation of ABI4 during this process. In addition, *35S*:*ABI4*^*Cys250Ala*^ *abi4 rbohd* transgenic plants showed ABA-hyposensitive phenotypes indicative of inactive ABI4. Compared with *35S*:*ABI4 abi4 rbohd*, H_2_O_2_-induced stomatal closure was partially inhibited in *35S*:*ABI4*^*Cys250Ala*^ *abi4 rbohd* plants, further supporting a critical role for Cys250-mediated sulfenylation in ABI4 activation.

Consistent with these physiological observations, analysis of ROS accumulation in the transgenic plants revealed that ABA-induced ROS levels correlated with the functional status of ABI4 sulfenylation (Fig. 4C). Collectively, these genetic and physiological data demonstrate that RBOHD-derived ROS regulate ABA signaling by promoting the sulfenylation of ABI4 at Cys250, thereby enabling ABI4-dependent transcriptional activation and stomatal closure in *Arabidopsis*.

### 2.5 Sulfenylation of ABI4 at Cys250 is critical for plant drought tolerance

**Fig. 5:**
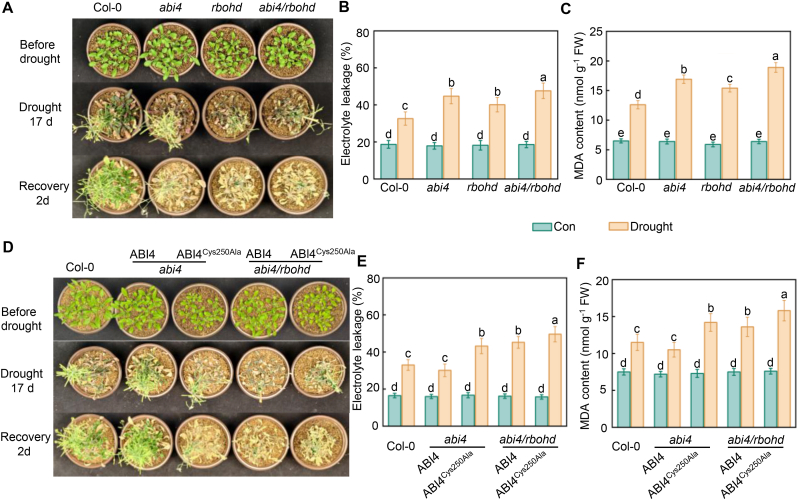
ABI4 regualtes plant drought tolerance through RBOHD-mediated Cys250 sulfenylation. **A** Drought tolerance phenotypes of Col-0, *abi4*, *rbohd*, and *abi4 rbohd* mutants and their related survival rate after recovery. **B–C** Relative electrolyte leakage and MDA content in plant leaves shown in (A**)**. **D** Drought tolerance phenotypes of transgenic lines expressing *ABI4* or *ABI4*^*Cys250Ala*^ in *abi4* or *abi4 rbohd* backgrounds and their related survival rate after recovery. **E-F** Relative electrolyte leakage and MDA content in plant leaves shown in (D**)**. Data represent means ± SE from at least three independent biological replicates. Different letters indicate statistically significant differences (*P* < 0.05, Duncan's multiple range test).

To further evaluate the physiological relevance of the sulfenylation of ABI4, we assessed the drought tolerance of *abi4*, *rbohd*, *abi4 rbohd*, *35S*:*ABI4 abi4, 35S*:*ABI4*^*Cys250Ala*^ *abi4, 35S*:*ABI4 abi4 rbohd*, and *35S*:*ABI4*^*Cys250Ala*^ *abi4 rbohd* plants. Compared to the wild type, drought tolerance was significantly weakened in *abi4*, *rbohd*, and *abi4 rbohd* plants (Fig. 5A). After the plants were rewatered, these mutants did not resume growth, with *abi4 rbohd* exhibiting the most severe wilting. Ion leakage and malondialdehyde (MDA) content assays indicated that drought caused more membrane injury in *abi4 rbohd* than in *abi4*, *rbohd*, and wild-type plants (Fig. 5B and C). These results suggest that ABI4 and RBOHD synergistically regulate plant drought tolerance. Moreover, the drought-sensitive phenotype of *abi4* was fully rescued by overexpressing *ABI4* but not *ABI4*^*Cys250Ala*^, suggesting that the sulfenylation of ABI4 at Cys250 is required for its role in the response of Arabidopsis to drought stress (Fig. 5D–F).

Previous ChIP-seq showed that many drought-responsive genes are the direct targets of ABI4 [24], including *DEHYDRATION-RESPONSIVE ELEMENT BINDING PROTEIN* (*DREB1A*, *DREB1D*, and *DREB2A*), *ABA RESPONSE ELEMENT-BINDING FACTOR* (*ABF2* and *ABF4*), dehydrin gene *RAB18*, and *NINE-CIS-EPOXYCAROTENOID DIOXYGENASE 3* (*NCED3*). We therefore investigated whether the expression of these genes would be affected by the sulfenylation of ABI4. Indeed, the drought-induced expression of these genes was significantly impaired in both *abi4* and *abi4 rbohd*, which was rescued by overexpressing *ABI4* but not *ABI4*^*Cys250Ala*^ (Fig. S2 and Fig. S3). Collectively, these results suggest that ABI4 and RBOHD synergistically regulate drought tolerance in *Arabidopsis* and that the sulfenylation of ABI4 at Cys250 is critical for this response.

## 3 Discussion

**Fig. 6:**
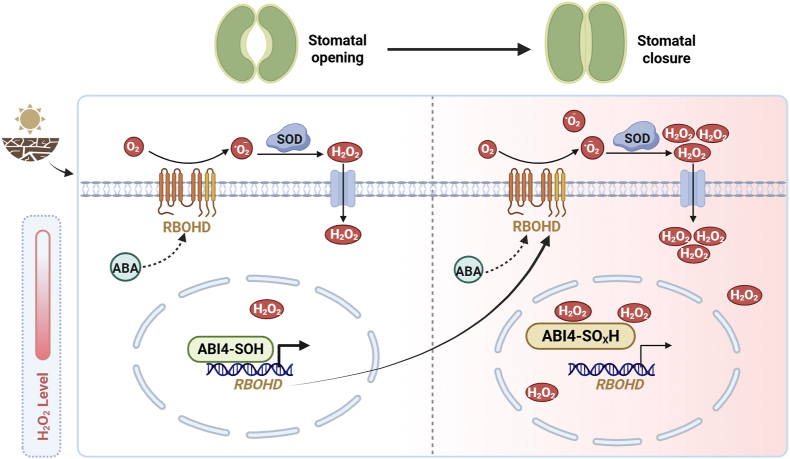
Working model shown in this study. Upon drought stress condition, ABA rapidly induces *RBOHD* expression and activation via ABI4, leading to transient H_2_O_2_ accumulation. This H_2_O_2_ pulse promotes sulfenylation of ABI4 at Cys250, thereby enhancing its DNA-binding affinity and transcriptional activity toward downstream target genes such as *RBOHD* itself and other drought-responsive genes. Prolonged or excessive H_2_O_2_ accumulation, however, results in ABI4 overoxidation and functional attenuation. The H_2_O_2_ -dependent inactivation of ABI4 under prolonged stress conditions may serve as an intrinsic braking mechanism to prevent excessive signaling.

In this study, we unraveled the molecular framework by which feedback regulation of ABI4 oxidation orchestrates ABA responses and drought tolerance by manipulating RBOHD-induced ROS production (Fig. 6). Following the perception of ABA signals, the activated expression of *RBOHD* and the resulting ROS production trigger stomatal closure. The master transcription factor ABI4 is required for ABA-induced *RBOHD* expression and ROS production. Furthermore, ROS production stimulates the oxidation of ABI4 at Cys250, which enhances its DNA-binding ability and transactivation activity towards *RBOHD*, thus forming a feedback loop to fine-tune the ABA signal transduction networks. Therefore, ABI4 is an oxidation target that links ROS and ABA signaling.

ROS function as key signals in both stress and plant hormonal responses [25], with the ABA-induced ROS burst serving as a prominent example. ABA-induced stomatal closure is dependent on ROS production, a process largely mediated by RBOHD and RBOHF [11]. The abundance of these RBOHs is regulated transcriptionally, with transcript levels increasing in response to ABA treatment or abiotic stresses such as drought and salinity [26,27]. In line with these observations, *RBOHD* transcript levels rapidly increase in response to ABA treatment, as does ROS production. However, little is known about the transcriptional regulatory mechanism of RBOHD, especially during ABA responses.

Here, we obtained multiple lines of evidence from genetic and physiological studies demonstrating that ABI4 is required for ABA-induced RBOHD activation and ROS production. This conclusion is supported by our observation that ABA-induced *RBOHD* upregulation and ROS accumulation were reduced in the *abi4* mutant. Salinity-induced ABI4 binds directly to the *RBOHD* promoter to enhance its transcription [21]. Our ChIP-qPCR and transactivation assays confirmed that the ABA-induced transcription of *RBOHD* is largely dependent on ABI4. Furthermore, our genetic analysis suggested that ABI4 acts upstream of ABA-induced RBOHD-dependent ROS production. H_2_O_2_ treatment induced full stomatal closure in *rbohd*, but not in *abi4* or *abi4 rbohd* plants, indicating that ROS-induced stomatal closure also requires functional ABI4. These observations highlight the intricate crosstalk between ROS signaling and ABI4-mediated networks.

In addition to transcriptional regulation, RBOH activity is also post-translationally regulated to coordinate the timing and magnitude of the ROS burst in regulating different biological processes [12,28,29]. Hydrogen sulfide (H_2_S) regulates ABA signaling in guard cells through the persulfidation of two cysteines in the C-terminus of RBOHD. Persulfidation helps RBOHD trigger a ROS burst, which in turn causes stomatal closure [12]. This might represent a mechanism underlying the rapid, ABA-triggered ROS production in guard cells. Thus, ABI4-mediated transcriptional regulation and H_2_S-triggered post-translational regulation compose a multi-layered, intricate mechanism for the regulation of RBOHD and ROS bursts that fine-tunes ABA signaling.

ROS exert their regulatory functions primarily through OxiPTMs of proteins [19]. In plants, H_2_O_2_ can react with particular Cys thiols to form S-sulfenylated residues. This redox regulation affects the redox states of many functional proteins and fine-tunes plant development and stress responses. However, the underlying mechanisms and physiological relevance of this process in plant ABA signaling are still poorly understood. In this study, we demonstrated the detailed mechanism by which ABI4-mediated regulation of ABA signaling is controlled by H_2_O_2_-induced oxidation. Immunoblotting combined with site-directed mutagenesis of cysteines indicated that ABI4 undergoes sulfenylation at Cys250 under H_2_O_2_ or ABA treatment. The sulfenylation level of ABI4 was correlated with changes in ROS levels and *RBOHD* transcript levels in response to ABA treatment, which both exhibited a peak pattern, indicating that the role of RBOHD in generating ROS involves the oxidation of ABI4. Further genetic analysis confirmed that RBOHD-produced ROS are responsible for ABA-triggered sulfenylation of ABI4 at Cys250 and that the sulfenylation of ABI4 is linked to ABA and drought-stress responses, as overexpression of *ABI4*^*Cys250Ala*^ failed to rescue the ABA hyposensitivity or drought-stress sensitivity of the *abi4* mutant. These results indicate that Cys250 functions as a redox-sensitive molecular switch that integrates ROS signals into ABA signaling and drought-stress responses.

We previously demonstrated that ABI4 undergoes persulfidation at Cys250, which is triggered by ABA in a time-dependent manner, and that this persulfidation is also essential for ABI4 activity and the H_2_S-mediated ABA signal relay [14]. Given that sulfenylation is required for persulfidation, these findings indicate that ROS and H_2_S act synergistically to fine-tune ABA signaling by regulating the different oxidative modifications of ABI4. Moreover, functional analyses using EMSA, ChIP-qPCR, and dual-luciferase assays revealed that this modification greatly affects the function of ABI4, including enhancing its DNA-binding ability and transactivation activity toward RBOHD. Thus, sulfenylation of ABI4 forms a functional feedback loop that facilitates ROS production and ABI4-mediated molecular networks in regulating stomatal closure and drought-stress responses. Such a positive feedback loop likely amplifies ABA signaling during the early phase of stress perception, ensuring a robust and timely response.

In the current study, we uncovered a redox-dependent regulatory mechanism in which RBOHD-derived H_2_O_2_ controls ABA signaling by modulating ABI4 activity through site-specific sulfenylation. This mechanism also provides a direct molecular link between ROS signaling and transcriptional regulation, offering insight into how plants integrate phytohormonal and redox cues to orchestrate adaptive responses. Given the widespread involvement of ROS and cysteine-based redox modifications in plant signaling, similar regulatory modules might operate in other plant hormone pathways and stress contexts. Further studies aimed at identifying additional redox-sensitive transcription factors and their regulatory mechanisms should advance our understanding of redox signaling networks in plants.

We also observed a decrease in the sulfenylation level of ABI4 after 30 min of ABA treatment. This might be attributed to the high H_2_O_2_ level that leads to the overoxidation of Cys, as ROS contents dramatically increased while the sulfenylation level of ABI4 decreased after 30 min of treatment. Overoxidation often inactivates protein activity or even causes protein degradation. Here, we demonstrated that the DNA-binding activity and transactivation activity of ABI4 significantly decreased after 30 min of ABA or H_2_O_2_ treatment (Fig. 3), which was highly correlated with the changes in its sulfenylation level. Thus, the ROS-dependent inactivation of ABI4 under prolonged stress conditions may serve as an intrinsic braking mechanism to prevent the continuous triggering of ABA signaling.

This dual regulatory mode, activation by moderate ROS levels and suppression by overoxidation, provides a conceptual framework for understanding how plants balance sensitivity and robustness in phytohormone signaling networks. A recent study demonstrated that the functions of the circadian clock transcription factor CCA1 HIKING EXPEDITION (CHE), which is required for both the basal circadian rhythm and pathogen-induced systemic salicylic acid production, are regulated by its oxidation status [30]. Higher ROS levels in local tissues induce the sulfinylation of CHE, which abolishes its binding to the salicylic acid biosynthesis gene *ISOCHORISMATE SYNTHASE1*, while lower ROS levels in distal tissues sulfenylate CHE, thereby enhancing its DNA-binding activity. Similarly, persulfidation and persulfide-oxidation regulate the function of RBOHD in mediating ABA signaling [12]. Since stomata are essential for gas exchange and water evaporation during photosynthesis and transpiration, the on-off switch for ABI4 function controlled by its oxidation status might help plants balance growth and stress responses by dynamically regulating stomatal movement.

## 4 Materials and methods

### 4.1 Plants materials and growth conditions

The Arabidopsis (*Arabidopsis thaliana*) *abi4* (SALK_103855; Col-0) mutant was obtained from the Arabidopsis Biological Resource Center (http://www.arabidopsis.org/abrc); *rbohd* was a gift from Prof. Kai Shu, School of Ecology and Environment, Northwestern Polytechnical University. The *abi4 rbohd* double mutant line was obtained by crossing *rbohd* with *abi4* (Table S1). Homozygous mutants were identified by sequencing, combined with PCR-based genotyping and corresponding phenotypes. Seeds were surface sterilized and washed three times with sterile water for 20 min, then cultured in Petri dishes on solid half-strength Murashige and Skoog (1/2 MS) medium (pH 5.8). Plants were grown in a growth chamber with a 16 h/8 h (23 °C/18 °C) day/night regime using bulb-type fluorescent light at a light intensity of 100 mmol photons m^−2^ s^−1^ irradiation. For drought tests, one-week-old seedlings grown on MS medium were transferred to the soil [31]. Transferred seedlings were adapted to the soil for one week under identical conditions followed by withholding water for 14 d. After rewatering for 1 d, recovery of WT, mutants and transgenic plants was monitored. Three experimental repeats were carried out each one containing at least 24 plants.

### 4.2 For expression in *Escherichia coli*

PCR-amplified full-length ABI4 was introduced into the pET28a vector (for His fusion) using a homologous recombination technique (Vazyme) with the enzyme digestion sites *Bam*HI and *Eco*RI. Site-directed mutagenesis was constructed previously and maintained in the lab [14]. The specific primers used for ABI4 are listed in Table S1. Expression and purification of the recombinant protein were performed in *BL21* cells (Vazyme). A quantity of isopropyl b-d-1-thiogalactopyranoside (0.1 mM) was added, and the bacteria were grown at an optical density at 600 nm of 0.4 to 0.6 for 12 h at 16 °C. After enriching the bacterial solution, the precipitate was suspended in PBS buffer, the protein was fragmented with an ultrasonic crusher, the mixture was centrifuged at 12,000 *g* for 30 min, and the extract was used for purification. The His-tagged proteins were purified using an NI-NTA prepacked gravity column (Sangon Biotech); the protein purification procedure was performed in accordance with the column specifications.

### 4.3 For transient expression in Arabidopsis protoplasts

PCR-amplified fragments were cloned into the PAN580 vector at the *Xba*I and *Bam*HI sites using a homologous recombination technique (Vazyme). *Arabidopsis* protoplasts were isolated according to the method described previously [32,33]. The transient expression vector PAN580 was delivered into *Arabidopsis* protoplasts using a PEG-calcium mediated approach, and protoplasts were cultured for 12 h. Protoplasts expressing the targeted GFP driven by the constitutive *35S* enhancer were identified by fluorescence microscopy and used for transient expression analysis.

### 4.4 For expression in planta

ABI4 products were cloned into the pCAMBIA 1305 vector (obtained from Aying Zhang's lab, College of Life Sciences, Nanjing Agricultural University). The constructed plasmids were transferred into an *Agrobacterium tumefaciens* strain and transformed into *Arabidopsis* using the floral dip method. Transgenic plants were selected on 1/2 MS medium containing Hygromycin B (50 mg/mL). Identification of transgenic plants was performed by genotyping combined with PCR, fluorescence observation, and immunoblot analysis.

### 4.5 SDS-PAGE and immunoblotting

Protein extracts were separated by 12% SDS-PAGE. After electrophoresis, the protein was transferred to a polyvinylidene difluoride membrane (Roche) at 100 V for 45 min at 4 °C. The membrane was blocked with 5% skimmed blocking solution (5% [w/v] BSA in TBS with 0.5% [v/v] Tween-20) and incubated for 2 h with shaking at room temperature or overnight at 4 °C. Immunoblot analysis was performed with antibodies. ImageJ (https://imagej.nih.gov/ij/) was used to quantify protein abundance, and signals from three independent experiments were quantified.

### 4.6 Immunochemical detection of sulfenylated proteins

Sulfenylated protein was detected in two different methods according to the method described previously [34,35]. Briefly, for *in vitro* assays, the extracted ABI4-GFP and its cystine mutant variations were treated with H_2_O_2_ at the indicated concentrations. The sulfenylated cysteines were labeled with dimedone and detected by immunoblotting with a 1:10,000 dilution of rabbit anti-Cys-SOH antibody (Millipore). For the *in vivo* assay, protoplasts expressing *35S:ABI4-GFP* and its cystine mutant variations were treated with H_2_O_2_ and ABA at the indicated concentrations and then collected by centrifugation. Sulfenylated proteins were analyzed by Western blotting using two complementary approaches: (1) cells were resuspended in dimedone lysis buffer (1 mM dimedone, 0.1% Triton X-100, 0.012 M Na_2_HPO_4_/0.003 M citric acid [pH 6]) for 20 min. The lysate was subsequently reconstituted in non-reducing SDS sample buffer containing 5 mM NEM. These samples purified by with GFP magnetic beads (Smart-Lifesciences) were run on an SDS-PAGE gel, and the protein was transferred onto a polyvinylidene difluoride membrane. Proteins were detected with a 1:10,000 dilution of rabbit anti-Cys-SOH antibody (Millipore); and (2) cells were resuspended in HEN lysis buffer (25 mM Hepes-NaOH, 1 mM EDTA, 0.1 mM Neocuproine, 0.1% Triton X-100 [pH 7.7]). Then the lysate was incubated at 50 °C for 20 min with 4 × volumes of blocking buffer (20 mM methyl-methanethiosulfonate, 2.5% SDS) to block the free thiols of the cysteines. The samples were then precipitated with 2 × volumes of ice-cold acetone overnight at −20 °C, after which the precipitated proteins were resuspended in HENS buffer (HEN buffer with 1% SDS). 2 mM Biotin-HPDP and 1 mM sodium arsenite were added into the suspensions and incubated at room temperature for 1 h to reduce –SOH to –SH, which subsequently reacts with Biotin-HPDP and labeled proteins containing –SOH groups. After the removal of excess Biotin-HPDP with acetone, the proteins were resuspended in HENS buffer, and 2 × volumes of neutralization buffer (20 mM Hepes-NaOH, pH 7.7, 1 mM EDTA, 100 mM NaCl, 0.5% Triton X-100) and GFP magnetic beads were added to bind the biotinylated proteins at 4 °C. Proteins were detected with a 1:10,000 dilution of anti-biotin-HRP (Abcam).

### 4.7 Measurement of ROS in protoplasts, seedings and guard cells and stomatal aperture

To detect ROS in protoplasts, protoplasts treated with or without ABA at the indicated concentrations were collected by centrifugation and then resuspended in W5 buffer (2 mM MES, 154 mM NaCl, 125 mM CaCl_2_, 5 mM KCl), loaded with 2′,7′-Dichlorodihydrofluorescein diacetate (H_2_DCF-DA, Sigma-Aldrich) for 15 min before washing in W5 buffer three times for 15 min each. A TCS-SP2 Laser Scanning Confocal Microscope was used to analyze the results (Leica; excitation at 488 nm, emission at 500 nm to 530 nm for the detection of ROS).

To detect ROS in *Arabidopsis* seedings, five-day-old seedlings of each genotype, cultured in 1/2 MS medium, were loaded with H_2_DCF-DA for 15 min before washing in MES buffer three times for 15 min each, subsequently treated with or without 10 μM ABA for 30 min and then analyzed using a TCS-SP2 Laser Scanning Confocal Microscope.

To detect ROS in guard cells, epidermal strips were soaked in opening buffer loaded with H_2_DCF-DA diacetate for 15 min before washing in MES buffer three times for 15 min each, subsequently treated with or without 10 μM ABA for 30 min and then analyzed using a TCS-SP2 Laser Scanning Confocal Microscope.

### 4.8 Stomatal aperture assays

Stomatal aperture measurements were carried out as described previously [23]. Epidermal strips were soaked in opening buffer containing 10 μM H_2_O_2_ or 10 μM ABA for 30 min. All manipulations were performed at 25 ± 1 °C. Data were analyzed by using ImageJ software. Each value represents the mean of at least 20 stomata taken from three independent experiments, and bars represent the SE of each treatment.

### 4.9 EMSA

The promoter region containing the CCAC motif was selected as the probe. The wild-type and mutated oligonucleotides (Table S1) for EMSA probes were biotin labeled using an EMSA Probe Biotin Labeling Kit (Beyotime) according to the manufacturer's instructions. DNA-protein binding reactions were carried out using an EMSA-Gel Shift Kit (Beyotime) at 25 °C according to the manufacturer's instructions. In parallel, competition experiments were performed with 10/100/200 times excess unlabeled wild-type or mutated probes to determine the specificity of the DNA-protein binding reactions. After 20 min of incubation, the completed reactions were separated by non-denaturing 4% PAGE, and the gel was subjected to autoradiography.

### 4.10 ChIP-qPCR analysis

Chromatin immunoprecipitation (ChIP) was essentially conducted as described previously [36]. In brief, 10-day-old *Arabidopsis* seedings were treated with 0.1% formaldehyde to cross-link the protein-DNA complexes. After isolation, the supernatants were incubated with GFP antibody (Abcam). Then a heat (65 °C) treatment was employed to reverse the protein-DNA complex. After protein degradation by proteinase K treatment and DNA precipitation by ethanol and purifcation, qPCR was used to quantify the enrichment with primers listed in Table S1. A region without CCAC motif from the *RBOHD* promoter was chosen as the negative control [21].

### 4.11 RT-qPCR analysis

Total RNA was isolated from the 10-day-old *Arabidopsis* seeding after ABA treatment at indicated time points using the TRIzol reagent (Vazyme) according to the manufacturer's instructions. qRT-PCR was performed using a Thermo Fisher Scientific Q5 Real-Time PCR System (Thermo Fisher Scientific) in a reaction mixture of 20 mL with ChamQ Universal SYBR qPCR Master Mix (Vazyme) according to the manufacturer's instructions. The specific primers for qPCR are listed in Table S1.

### 4.12 Dual-LUC reporter system

The pGreenII 0800-LUC vector carrying the promoter was used as a reporter plasmid as described previously [37]. For the effector plasmids, the TF coding sequences were inserted into the PAN580 vector. *Arabidopsis* protoplasts were extracted from 15-day-old seedlings as described above. The effector plasmid and reporter plasmid were combined in equal amounts and transformed into the protoplasts. Cells were cultured at 22 °C for 12 h. In the control group, the empty PAN580 vector replaced the effector plasmid at the same concentration. The activities of firefly (*Photinus pyralis*) and Renilla (*Renilla reniformis*) LUCs from a single sample were measured sequentially using the dual-LUC reporter assay system (Beyotime), and the values were recorded using an iD5-1 microplate reader (Molecular Devices). The ratio of firefly to Renilla LUC activity was used as an indicator of transcriptional efficiency. More than three biological replicates were performed.

### 4.13 MDA and electrolyte leakage assay

The MDA content and the percentage of electrolyte leakage were determined from *Arabidopsis* seedling leaves with or without drought treatment for 10 d according to previously reported methods [38,39].

### 4.14 Statistical analysis

Statistical analysis and graph construction were performed using SPSS 22.0 (https://www.ibm.com/products/spss-statistics). Differences were considered significant at *P* < 0.001, 0.01, or 0.05.

## CRediT authorship contribution statement

**Zhenglin Ge:** Writing – original draft, Investigation, Formal analysis, Data curation. **Tiantian Wu:** Writing – original draft, Methodology, Investigation, Formal analysis, Data curation. **Zhengyao Lin:** Writing – original draft, Investigation, Data curation. **Sisong Lai:** Formal analysis, Data curation. **Guojing Chen:** Formal analysis, Data curation. **Lihui Xiao:** Formal analysis, Data curation. **Jing Zhang:** Data curation. **Kailu Zhang:** Formal analysis. **Heng Zhou:** Writing – review & editing, Project administration, Formal analysis, Data curation, Conceptualization. **Yanjie Xie:** Writing – review & editing, Supervision, Project administration, Conceptualization.

## Declaration of competing interest

The authors declare that they have no known competing financial interests or personal relationships that could have appeared to influence the work reported in this paper.

## Figures and Tables

**figs1:**
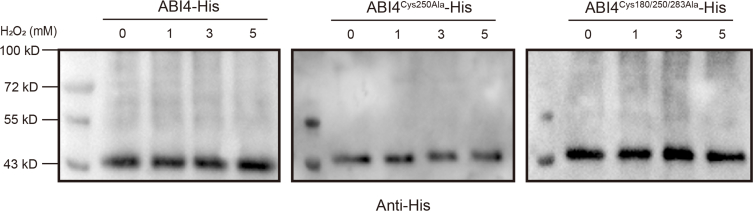


**figs2:**
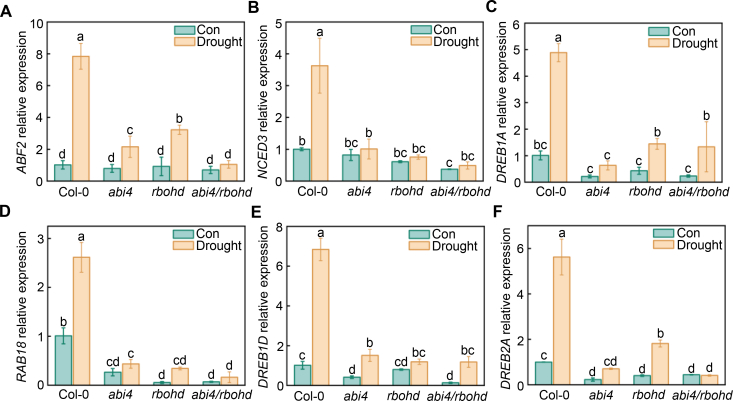


**figs3:**
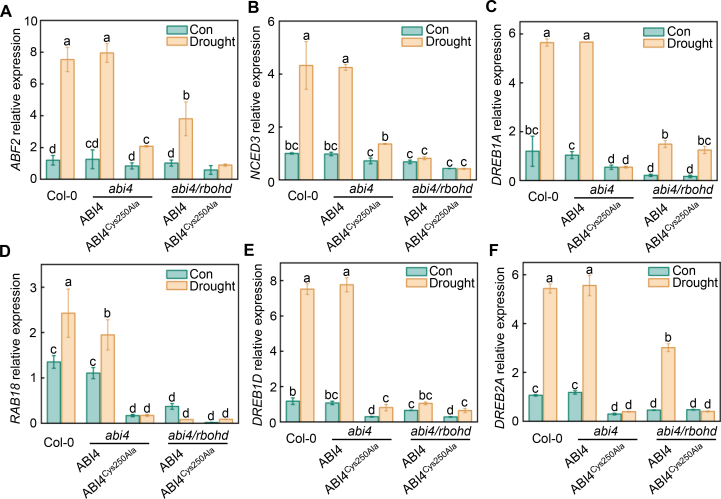


## Data Availability

All data supporting the findings of this study are available within the paper and its Supplementary Information.
